# Down syndrome burden in China and globally: a comparative analysis of 1990–2021 trends and future projections based on GBD 2021 database

**DOI:** 10.3389/fpubh.2025.1632250

**Published:** 2025-08-11

**Authors:** Xiangwen Tu, Feng Zhang, Junkun Chen, Manmei Tang

**Affiliations:** ^1^Laboratory of Eugenics Genetics, Ganzhou Women and Children's Health Care Hospital, Ganzhou, Jiangxi, China; ^2^Department of Pediatric Neurological Rehabilitation, Ganzhou Women and Children's Health Care Hospital, Ganzhou, Jiangxi, China; ^3^Department of Laboratory, Ganzhou Center for Disease Control and Prevention, Ganzhou, Jiangxi, China

**Keywords:** bayesian age-period-cohort model, Down syndrome, global disease burden, joinpoint regression, socio-demographic index

## Abstract

**Background:**

Down syndrome (DS), a neurodevelopmental disorder caused by a chromosomal abnormality, poses a major burden on global health. Analyzing the disease burden of DS, both in China and globally, is crucial for refining public health strategies.

**Methods:**

Using the Global Burden of Disease (GBD) 2021 database, we examined age-standardized incidence rate (ASIR), age-standardized prevalence rate (ASPR), age-standardized mortality rate (ASMR), and age-standardized disability-adjusted life year rate (ASDR) for DS in China and globally from 1990 to 2021. Joinpoint regression analysis was applied to identify temporal trends by calculating the annual percent change (APC) and average annual percent change (AAPC). A bayesian age-period-cohort (BAPC) model was further employed to project prevalence changes from 2022 to 2036.

**Results:**

From 1990 to 2021, China’s ASIR decreased from 1.68 per 100,000 to 1.18 per 100,000, compared to a global reduction from 1.27 per 100,000 to 0.97 per 100,000. Similarly, ASPR in China fell from 28.01 per 100,000 to 24.8 per 100,000, while globally it dropped from 27.98 per 100,000 to 21.07 per 100,000. Notably, China experienced steeper declines in ASMR (EAPC = −4.18%) and ASDR (EAPC = −3.87%) compared to the global averages (−0.44% and −0.69%, respectively). Joinpoint regression analysis shows that from 1990 to 2021, China’s ASIR (AAPC = −1.15, *p* < 0.001), ASPR (AAPC = −0.39, *p <* 0.001), ASDR (AAPC = −2.87, *p* < 0.001), and ASMR (AAPC = −3.08, *p* < 0.001) for DS all decreased. The SDI was negatively correlated with ASMR (*R* = −0.68, *p <* 0.001) and ASDR (*R* = −0.66, *p* < 0.001) but positively associated with ASIR (*R* = 0.55, *p* < 0.001) and ASPR (*R* = 0.80, *p* < 0.001). Projections from the BAPC model suggest that the ASPR of DS will continue to decline both in China and globally through 2036.

**Conclusion:**

From 1990 to 2021, the disease burden of DS declined in China and globally. China’s decline in ASMR and ASDR outpaced the global level, though ASIR and ASPR remained higher. To further reduce DS burden, future efforts should prioritize early identification, counseling for informed decision-making, and equitable access to quality lifelong multidisciplinary support for affected individuals.

## Introduction

Down syndrome (DS), a congenital neurodevelopmental disorder resulting from trisomy 21 (1), ranks among the most prevalent chromosomal abnormalities worldwide, with an estimated incidence of 1 in 1,000 to 1 in 1,100 live births (2). This condition arises from chromosomal nondisjunction during cell division, leading to systemic developmental anomalies (3). Clinically, DS is characterized by distinctive craniofacial features, hypotonia, and mild to moderate intellectual disability. Approximately 40–50% of individuals with DS develop congenital heart defects, while others face elevated risks for childhood leukemia, gastrointestinal malformations, and early-onset Alzheimer’s disease (4). Over the past few decades, the epidemiology of DS has evolved significantly due to advancements in prenatal screening, diagnostic techniques, and medical care (5). Although early intervention programs and rehabilitation training can enhance functional outcomes (6), the lifelong need for medical attention and social support imposes substantial healthcare burdens and socioeconomic pressures on families and communities.

The GBD database serves as a vital resource that consolidates diverse data sources to estimate disease burden metrics, such as incidence, prevalence, disability-adjusted life years (DALYs), and mortality, offering a comprehensive picture of DS’s impact both globally and regionally (7). Previous studies, supported by GBD data, have documented a downward trend in the global incidence and prevalence of DS between 1990 and 2019 (8). Meanwhile, improvements in medical care and early interventions have enhanced both life expectancy and quality of life for individuals with DS (9). However, the global burden of DS remains considerable, with notable disparities in disease burden and healthcare outcomes across regions and socioeconomic levels (10). As the world’s largest middle-income country, China demonstrates unique epidemiological patterns of DS (11). The complex interaction between public health policies, demographic features, societal development stages, and cultural influences poses distinctive challenges for DS prevention and control in China (12). Although prior research has examined the disease burden of DS globally from 1990 to 2019, analyses leveraging the 2021 GBD data to assess China’s DS burden in the context of global trends remain scarce.

This study utilizes the GBD database to evaluate the disease burden of DS in China and globally, offering data that may inform mechanistic and interventional research. We compare trends in ASIR, ASPR, ASDR, and ASMR across China, the global population, and five SDI regions. BAPC models are then employed to forecast future trajectories of the DS burden. These findings are intended to equip policymakers and healthcare planners with actionable insights for designing targeted interventions and to provide descriptive background that may facilitate future investigations into prenatal and early-life influences on childhood health outcomes in DS populations.

## Method

### Data sources and study population

This study utilized the GBD 2021 dataset,^1^ in accordance with the GBD protocol.^2^ This dataset encompasses disease burden estimates across 204 countries and territories, covering 371 diseases and injuries, 88 risk factors, and 288 causes of death. We extracted age-standardized epidemiological metrics for DS, including incidence, prevalence, mortality, DALYs, years lived with disability (YLDs), and years of life lost (YLLs), from 1990 to 2021 across three geographical levels: global, China-specific, and five SDI regions. Details are in Supplementary material 1.

The SDI, a composite indicator developed by the GBD collaborator group, quantifies regional development status through three dimensions: per capita income, average educational attainment, and total fertility rate. This index ranges from 0 (lowest) to 1 (highest), with the global population categorized into five SDI quintiles: low (<0.466), low-middle (0.466–0.619), middle (0.619–0.712), high-middle (0.712–0.810), and high (≥0.810). China’s 2021 SDI value of 0.722 places it within the high-middle SDI category (13). The spearman rank correlation test was employed to evaluate associations between ASIR, ASPR, ASDR, ASMR, and SDI. Correlation coefficients and corresponding *p*-values were calculated, with significance set at *p* < 0.05.

All data were obtained from the publicly available GBD 2021 repository, which includes anonymized population-level statistics without individual identifiers. As the original studies contributing to the dataset received ethical approvals from respective institutional review boards, this secondary analysis of aggregated data is exempt from additional ethical review under international guidelines.

### Estimation and trend analysis

Based on the standard population from GBD 2021, we estimated the incidence cases, prevalence cases, deaths, and DALYs of DS from 1990 to 2021, along with ASIR, ASPR, ASMR, and ASDR, with corresponding 95% uncertain intervals (UIs). A regression model was employed to calculate the estimated annual percentage change (EAPC), a widely used metric for summarizing long-term trends in age-standardized rates (ASRs) of DS burden. The EAPC was derived using the formula: Y = *α* + *β*X + *ε*, where X represents the calendar year, Y denotes the natural logarithm of ASR, and ε is the error term. The EAPC was calculated as 100 × (exp(β) − 1). An upward trend was indicated if both the EAPC value and the lower bound of the 95% CI were greater than 0; a downward trend if both the EAPC value and the upper bound of the 95% CI were less than 0; and a stable trend if the 95% CI included 0.

### Joinpoint regression analysis

We used joinpoint regression to analyze temporal trends in DS burden indicators (ASIR, ASPR, ASMR, ASDR) in China and globally, calculating APC, AAPC, and 95% CIs. The model assumed poisson-distributed rates and piecewise linear trends. Optimal joinpoints (0–5) were selected via the Hudson algorithm, minimizing squared residuals, with the best-fit model determined by bayesian information criterion. Significance was assessed using monte carlo permutation tests (*p* < 0.05). APC indicated segment-specific trends, while AAPC summarized the 1990–2021 average. Trends were classified as declining (95% CI upper bound < 0), increasing (lower bound > 0), or stable. This method objectively identifies joinpoints while controlling overfitting.

### BAPC modeling approach for DS ASPR projections

The BAPC framework was used to forecast DS ASPR from 2022 to 2036. Age-stratified historical data (1990–2021, at 5-year intervals) were sourced from national registries and adjusted using capture-recapture methods. The model assumes that temporal trends arise from additive age effects, period effects, and cohort effects. Second-order random walk priors were applied to smooth age, period, and cohort dynamics, while weak gamma priors (shape = 1, rate = 0.01) minimized subjective assumptions. Posterior estimation was conducted using integrated nested laplace approximations (INLA), offering greater computational efficiency than traditional MCMC methods (14). Model validation included out-of-sample testing with data from 2015 to 2021. Probabilistic forecasts with 95% credible intervals were generated using the R BAPC package, incorporating United Nations demographic projections.

### Statistical analysis

Data organization was performed using Microsoft Excel 2021. Trend analysis of ASRs was conducted using Joinpoint Regression Program (Version 4.9.1.0) to calculate APC and AAPC (15, 16), with the significance level (*α*) set at 0.05. Statistical analyses and graphical representations were generated using R software (version 4.4.0).

## Result

### Burden and temporal trends of DS in China and globally from 1990 to 2021

According to Table 1, both China and the global population saw declines in DS incidence, prevalence, deaths, and DALYs. However, in 1990, China had higher ASIR and ASPR of DS compared to the global average. For example, in 1990, China had an ASIR of 1.68 per 100,000 people, while the global ASIR was 1.27 per 100,000. By 2021, China’s ASIR had decreased to 1.18 per 100,000, while the global ASIR had decreased to 0.97 per 100,000. The EAPCs for China and the global population were −1.21 and −0.82, respectively. Similarly, ASPR also declined in both China and globally, although the decline was less pronounced in China. In 1990, China had an ASPR of 28.01 per 100,000, which decreased to 24.8 per 100,000 in 2021, with an EAPC of −0.17. Globally, the ASPR decreased from 27.98 to 21.07 per 100,000, with an EAPC of −0.84. Both deaths and DALYs also decreased significantly in China and globally. China’s ASMR dropped from 0.63 to 0.23 per 100,000, and its ASDR decreased from 58.59 to 23.3 per 100,000, with EAPCs of −4.18 and −3.87, respectively. Globally, ASMR and ASDR decreased from 0.51 to 0.42 per 100,000 and from 45.49 to 35.2 per 100,000, with EAPCs of −0.44 and −0.69, respectively. Furthermore, both YLLs and YLDs also showed declines. In China, the age-standardized YLL rate decreased substantially from 55.56 per 100,000 in 1990 to 20.61 per 100,000 in 2021, with an EAPC of −4.20, while the YLD rate declined modestly from 3.03 to 2.69 per 100,000, with an EAPC of −0.17. Globally, YLLs decreased from 42.45 to 32.91 per 100,000 (EAPC: −0.68), and YLDs decreased from 3.04 to 2.30 per 100,000 (EAPC: −0.83), indicating a more pronounced reduction in YLLs compared to YLDs, particularly in China. Details are in Supplementary material 2.

**Table 1:** Burden of DS in China and globally: all-ages cases, age-standardized rates, and estimated annual percentage changes (1990–2021).

Location	Measure	1990		2021		EAPC (95%CI)
		All-ages cases (95%UI)	Age-standardized rates per 100,000 people (95%UI)	All-ages cases (95%UI)	Age-standardized rates per 100,000 people (95%UI)	
China	Incidence	18,578 (15,099, 22,437)	1.68 (1.37, 2.03)	6,250 (5,098, 7,568)	1.18 (0.96, 1.43)	−1.21 (−1.35, −1.06)
	Prevalence	358,968 (287,151, 444,274)	28.01 (22.42, 34.64)	239,365 (193,407, 294,130)	24.8 (19.96,30.39)	−0.17 (−0.36, 0.03)
	Deaths	6,934 (4,479, 11,125)	0.63 (0.40, 1.01)	1,424 (765, 1999)	0.23 (0.12, 0.33)	−4.18 (−4.59, −3.77)
	DALYs	654,145 (436,030, 1,028,444)	58.59 (38.82, 92.38)	149,515 (89,980, 203,243)	23.3 (13.27, 32.03)	−3.87 (−4.24, −3.5)
	YLLs	615,314 (396,994, 988,819)	55.56 (35.84, 89.39)	123,533(65,913, 174,606)	20.61 (10.82, 29.24)	−4.20 (−4.61, −3.79)
	YLDs	38,832 (25,778, 54,879)	3.03 (2.01, 4.27)	25,981 (17,548, 38,004)	2.69 (1.82, 3.94)	−0.17 (−0.36,-0.03)
Global	Incidence	81,676 (67,591, 99,173)	1.27 (1.05, 1.55)	59,807 (50,578, 69,714)	0.97 (0.82, 1.13)	−0.82 (−0.95, −0.68)
	Prevalence	1,658,220 (1,374,357, 2,006,034)	27.98 (23.22, 33.76)	1,566,169 (1,314,520, 1,856,978)	21.07 (17.65, 24.98)	−0.84 (−0.99, −0.69)
	Deaths	31,009 (17,462, 70,932)	0.51 (0.29, 1.15)	29,265 (22,458, 45,335)	0.42 (0.32, 0.68)	−0.44 (−0.55, −0.33)
	DALYs	2,803,958 (1,601,881, 6,346,043)	45.49 (26.17,102.27)	2,353,048 (1,759,096, 3,754,934)	35.2 (26.01, 57.17)	−0.69 (−0.78, −0.59)
	YLLs	2,624,101 (1,428,109, 6,145,196)	42.45 (23.30, 98.87)	2,181,832 (1,595,580, 3,590,813)	32.91 (23.74, 54.96)	−0.68 (−0.79, −0.56)
	YLDs	179,857 (122,383, 2,507,589)	3.04 (2.07, 4.24)	171,216 (117,695, 237,282)	2.30 (1.58, 3.19)	−0.83 (−0.97, −0.68)

CI: confidence interval, DS: Down syndrome, DALYs: disability-adjusted life years, EAPC: estimated annual percentage change, UI: uncertain interval, YLDs: years lived with disability, YLLs: years of life lost.

### Joinpoint regression analysis of the disease burden of DS in China and globally

**Figure 1:**
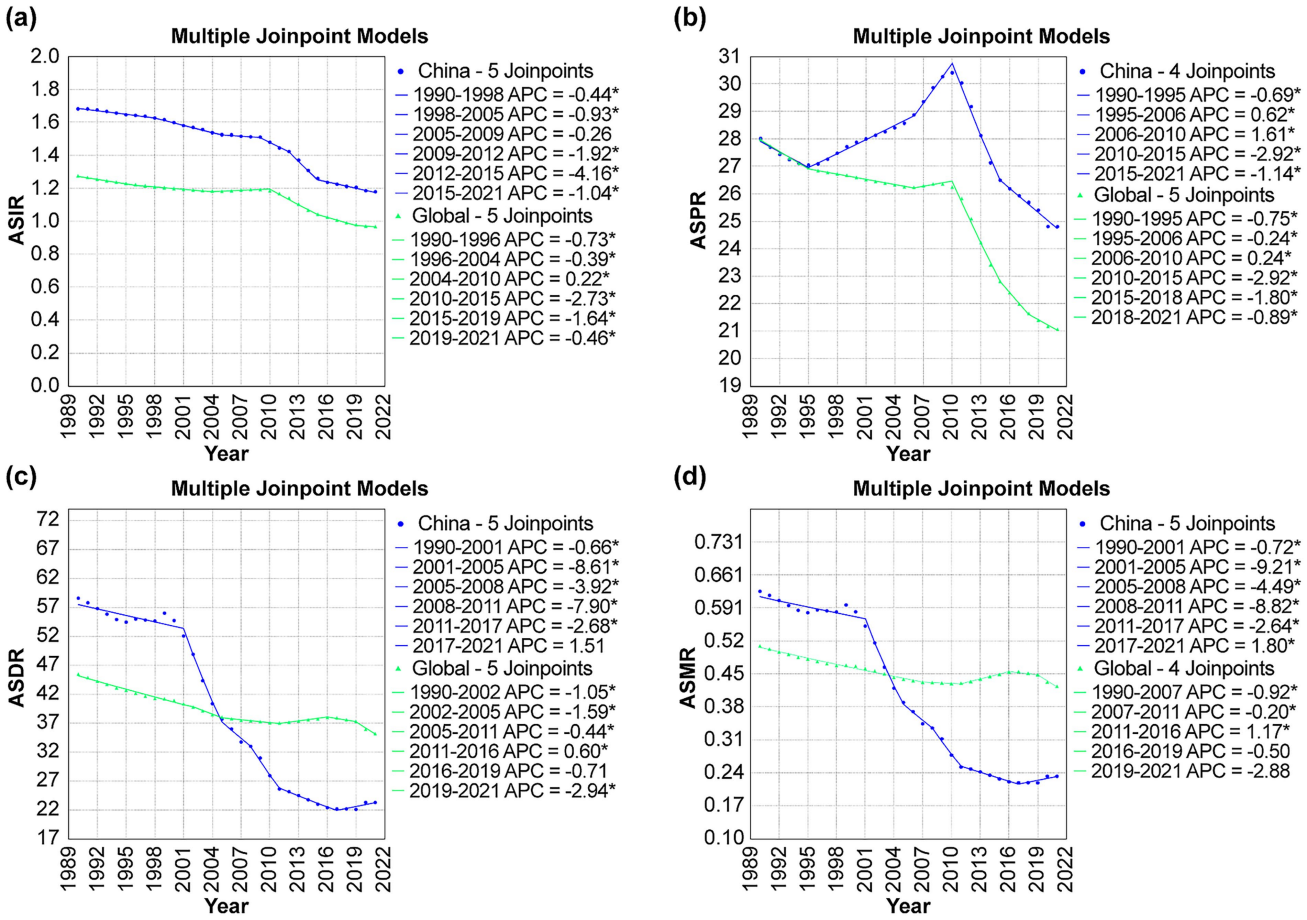
Joinpoint analysis of disease burden of DS in China and globally, 1990–2021: **(a) ASIR**, **(b) ASPR**, **(c) ASDR**, and **(d) ASMR**. APC: annual percentage change; ASIR: age-standardized incidence rate; ASPR: age-standardized prevalence rate; ASMR: age-standardized death rate; ASDR: age-standardizeddisability-adjusted life year rate; DALYs: disability-adjusted life years.

As shown in Figure 1a, the ASIR for DS exhibited declines in both China and globally. In China, the most significant declines were observed between 2009 and 2012 (APC = −1.92, *p* < 0.001) and 2012–2015 (APC = −4.16, *p* < 0.001). Globally, the decline was more gradual, with substantial reductions between 2010 and 2015 (APC = −2.73, *p* < 0.001) and 2015–2019 (APC = −1.64, *p* < 0.05). As depicted in Figure 1b, the ASPR for DS decreased in both China and globally. In China, the decline was temporarily interrupted by significant increases between 1995 and 2006 (APC = 0.62, *p* < 0.05) and 2006–2010 (APC = 1.61, *p* < 0.001), followed by a sharp decline between 2010 and 2015 (APC = −2.92, *p* < 0.001). Globally, the ASPR demonstrated a more consistent decline, with marked reductions between 2010 and 2015 (APC = −2.92, *p* < 0.001) and 2015–2018 (APC = −1.80, *p* < 0.001). As illustrated in Figure 1c, the ASDR for DS in China showed a consistent downward trend throughout the study period, with the most pronounced decreases occurring between 2001 and 2005 (APC = −8.61, *p <* 0.001) and 2008–2011 (APC = −7.90, *p* < 0.001). Globally, the ASDR also decreased, albeit more gradually, with significant reductions between 2002 and 2005 (APC = −1.59, *p* < 0.05) and 2019–2021 (APC = −2.94, *p* < 0.05). As shown in Figure 1d, both China and the global dataset revealed a long-term decline in ASMR for DS. China experienced the most substantial reductions between 2001 and 2005 (APC = −9.21, *p* < 0.001) and 2008–2011 (APC = −8.82, *p* < 0.001). Globally, the decline was steady but less pronounced, with significant reductions between 1990 and 2007 (APC = −0.92, *p* < 0.05) and 2019–2021 (APC = −2.88, *p* < 0.05). From 1990 to 2021, China saw a downward trend in ASIR (AAPC = −1.15), ASPR (AAPC = −0.39), ASDR (AAPC = −2.87), and ASMR (AAPC = −3.08) for DS. The declines in ASDR and ASMR were much steeper than the global ASDR (AAPC = −0.81) and ASMR (AAPC = −0.58) reductions. Globally, all these metrics also decreased, but to a smaller extent than in China. The results of AAPC shown in Table 2.

**Table 2:** AAPC of DS in China and globally from 1990 to 2021.

Measure name	AAPC(95%CI)	
	China	Global
ASIR	−1.15 (−1.24 – −1.06)	−0.89 (−0.94 – −0.83)
ASPR	−0.39 (−0.48 – −0.30)	−0.91 (−0.96 – −0.87)
ASDR	−2.87 (−3.33 – −2.41)	−0.81 (−0.96 – −0.66)
ASMR	−3.08 (−3.57 – −2.59)	−0.58 (−0.74 – −0.43)

AAPC: average annual percent change; ASIR: age-standardized incidence rate; ASPR: age-standardized prevalence rate; ASMR: age-standardized death rate; ASDR: age-standardized disability-adjusted life year rate; CI: confidence interval.

### Comparison of disease burden of DS among China, globally and SDI regions

**Figure 2:**
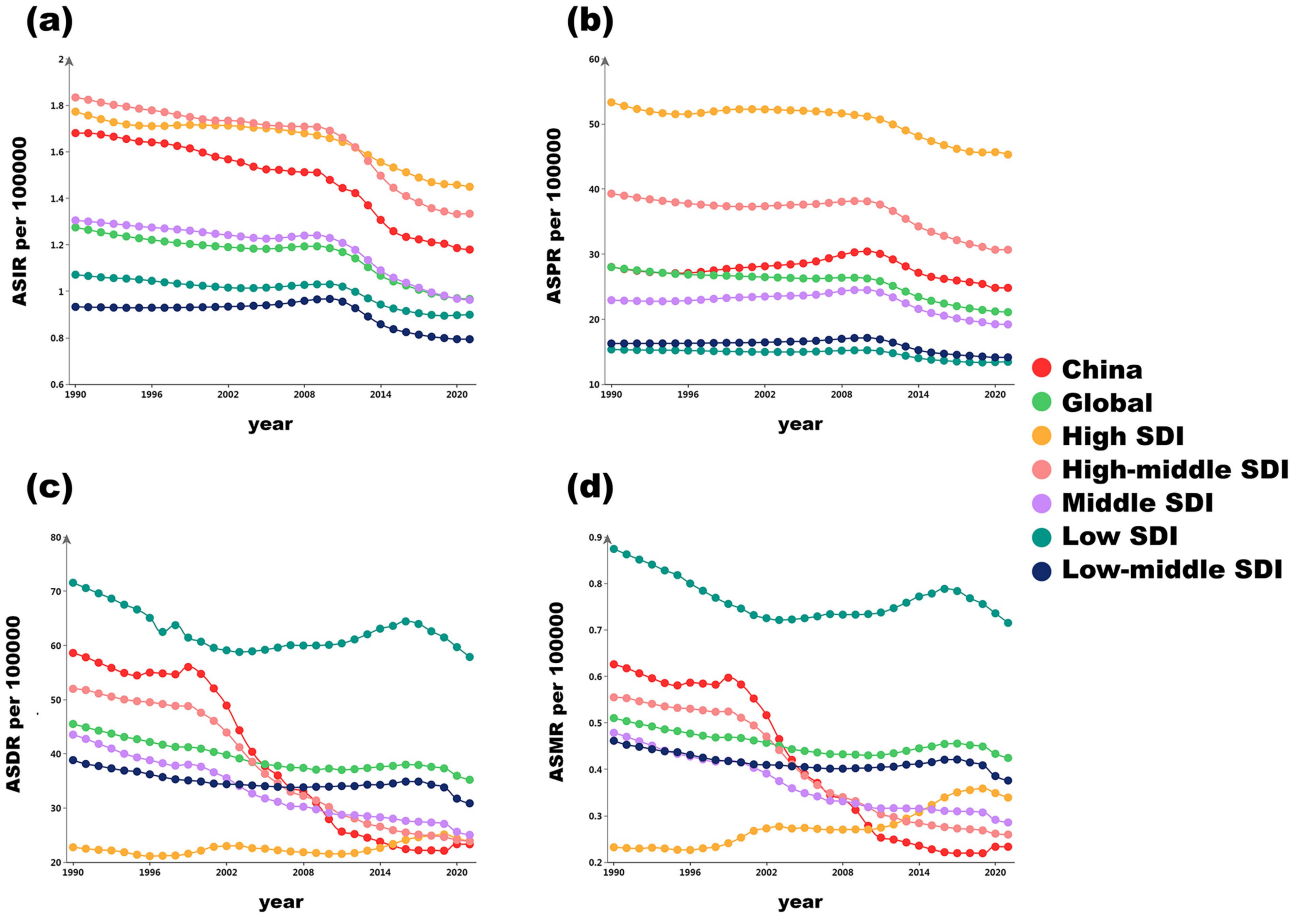
Trends in age-standardized rates of disease burden indicators for China, globally, and across different SDI regions from 1990 to 2021. **(a)** Trends in ASIR; **(b)** Trends in ASPR; **(c)** Trends in ASDR; **(d)** Trends in ASMR. ASIR: age-standardized incidence rate, ASPR: age-standardized prevalence rate, ASMR: age-standardized death rate, ASDR: age-standardized disability-adjusted life year rate, DALYs: disability-adjusted life years, SDI: social-demographic index.

From 1990 to 2021, low SDI areas showed the highest ASDR and ASMR, with ASDR (57.85) 2.4 times that of high SDI (23.86) and ASMR 3.3 times (0.71 vs. 0.34). China’s specificity in the high-medium SDI group, ASDR was lower than the same group, but ASIR was higher than the global. ASIR of DS in China and the world generally showed a downward trend, but fluctuated slightly from 2005 to 2010. ASPR showed a steady and slow decline. Compared with the world, the ASMR and ASDR of Chinese DS showed a significant downward trend, reaching 60.2 and 63.5%, respectively, far higher than the global averages (22.6 and 17.6%). The specific change trend is shown in Figure 2, Details are in Supplementary material 3.

### Association between ASRs of DS and SDI in 21 regions from 1990 to 2021

**Figure 3:**
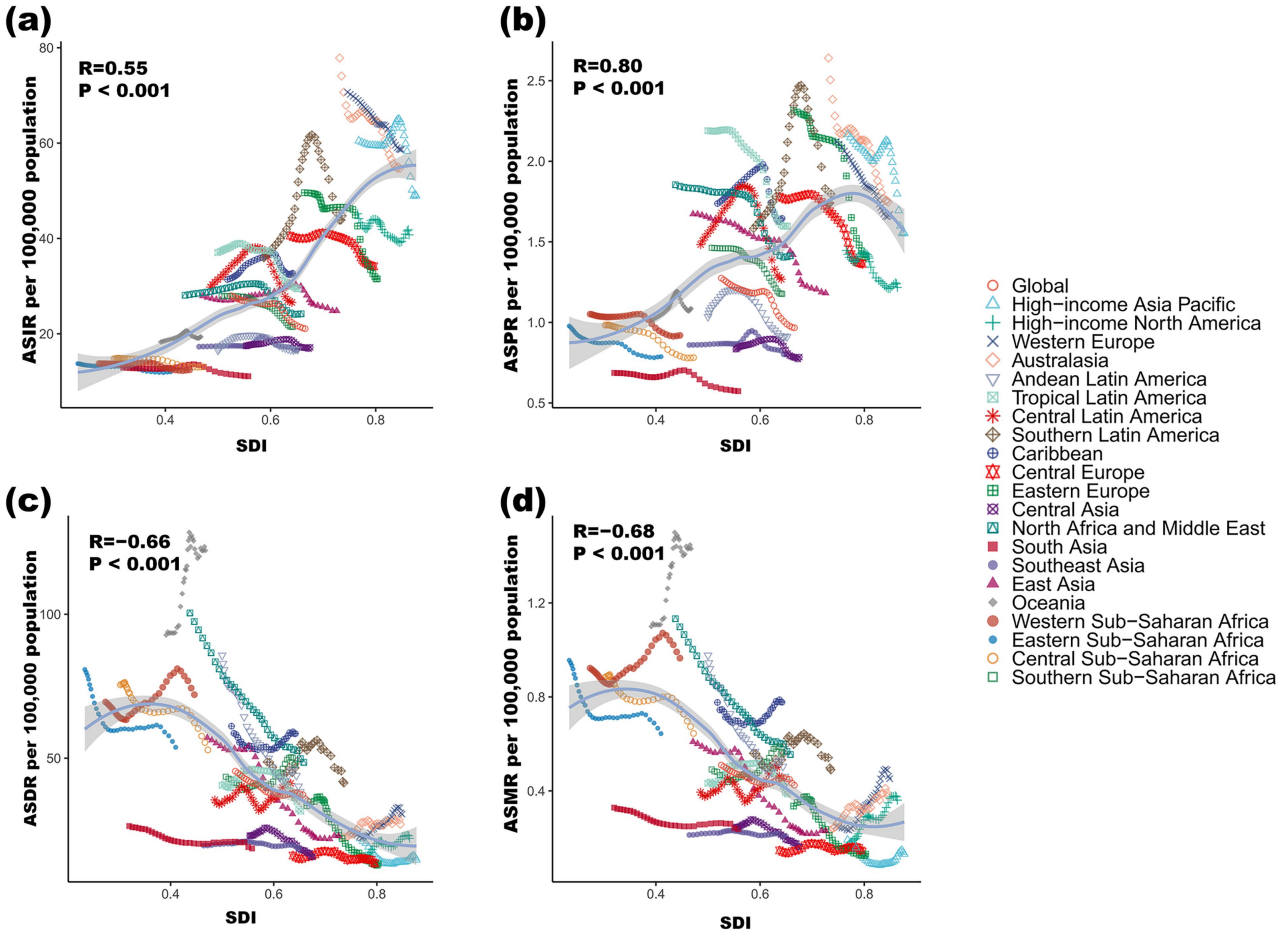
Association between ASIR **(a)**, ASPR **(b)**, ASDR **(c)**, and ASMR **(d)** of DS and SDI in 21 regions from 1990 to 2021. ASIR: age-standardized incidence rate, ASPR: age-standardized prevalence rate, ASMR: age-standardized death rate, ASDR: age-standardized disability-adjusted life year rate, DALYs: disability-adjusted life years, SDI: social-demographic index.

A cross-regional comparison of 21 GBD regions globally from 1990 to 2021 revealed that the ASIR and ASPR showed a positive correlation with the SDI. However, within the same region, increases in SDI were associated with decreases in ASIR and ASPR (Figures 3a,b). Overall, the ASMR and ASDR demonstrated a negative correlation with SDI. Notably, Oceania had significantly higher ASDR and ASMR values than expected, while Central Europe and the High-income Asia Pacific region had significantly lower values (Figures 3c,d). Globally, the overall correlation trend between ASMR and ASDR with SDI remained consistent. Yet, the correlations between ASDR, ASMR, and SDI exhibited significant variation across different regions. For instance, in low-SDI countries, ASDR and ASMR decreased as SDI increased. In contrast, some regions, such as Oceania and Western Sub-Saharan Africa, displayed a clear upward trend in these rates (Figure 3).

### Prediction of the prevalence of DS by gender in China and globally from 2021 to 2036

**Figure 4:**
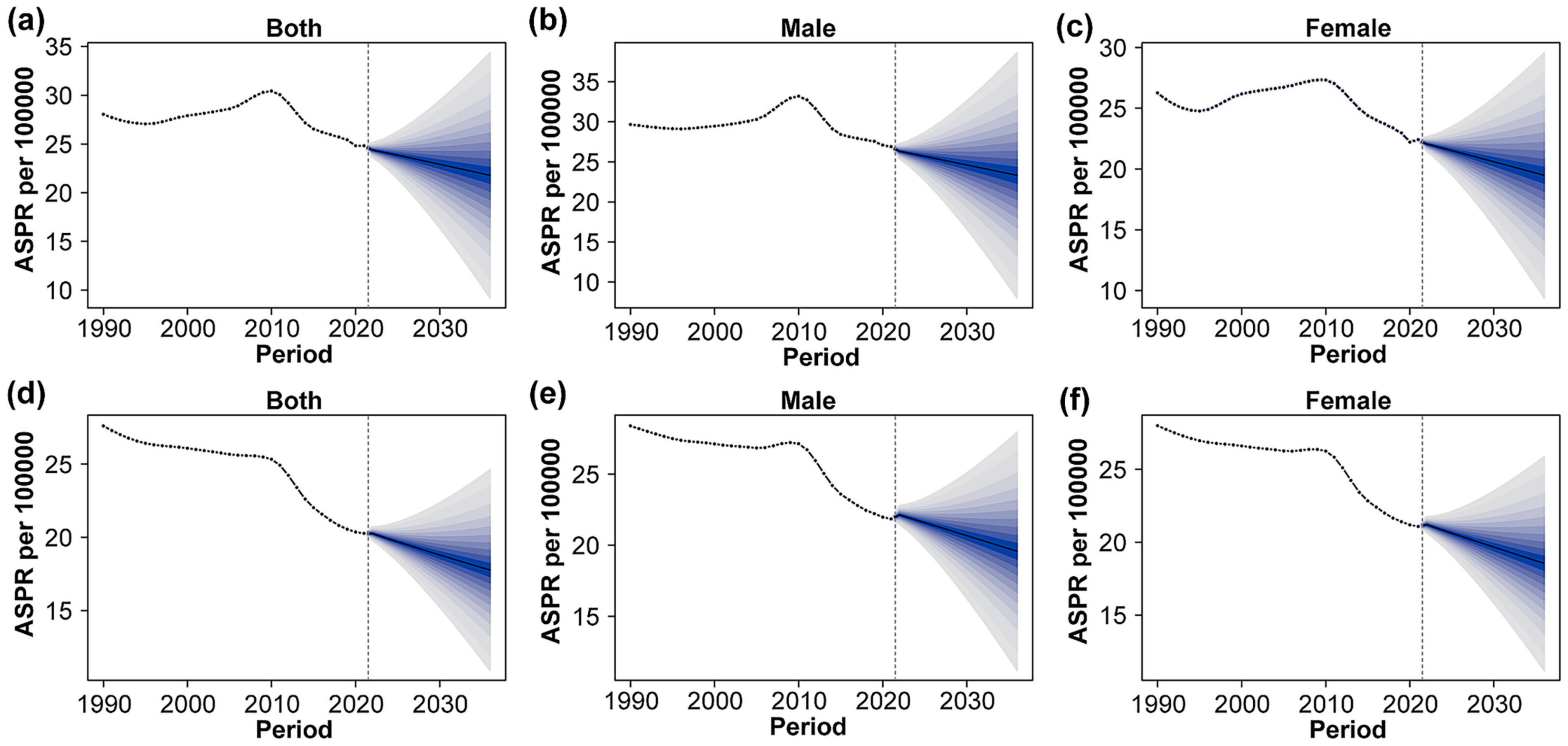
Trends and projections of ASPR for full sex **(a)**, male **(b)**, and female **(c)** in China and for full sex **(d)**, male **(e)**, and female **(f)** in the global DS from 1990 to 2035. Solid points and solid lines indicate the observed values, and the vertical dashed line indicates the position at which the prediction starts. Blue shading indicates the 95% highest density interval of the predicted value, as predicted by the BAPC analysis. ASPR: age-standardized prevalence rate.

From 2021 to 2036, the predicted ASPR of DS show a general declining trend for both China and globally across all genders (Figure 4). In China, the ASPR for both sexes is projected to decrease from 24.80 to 21.77, with male rates slightly higher than female rates each year. Similarly, globally, the ASPR for both sexes is expected to drop from 21.08 to 18.54. Throughout the projection period, male ASPRs exceed female ASPRs in all regions, Table 3. These projections highlight the anticipated decrease in DS prevalence over the next decade and a half. Details are in Supplementary materials 4, 5.

**Table 3:** Projected ASPR for DS in China and globally by gender.

Location name	Gender	1990 year	2021 year	2036 year
China ASPR per 100,000 people (95%CI)	both	28.03 (27.94, 28.12)	24.80 (24.70, 24.90)	21.77 (9.18, 34.36)
	male	29.67 (29.54, 29.80)	26.88 (26.74, 27.03)	23.32 (8.01, 38.63)
	female	26.25 (26.12, 26.37)	22.39 (22.25, 22.52)	19.48 (9.38, 29.57)
global ASPR per 100,000 people (95%CI)	both	28.01 (27.96, 28.05)	21.08 (21.05, 21.11)	18.54 (11.20,25.88)
	male	28.39 (28.33, 28.45)	21.84 (21.80, 21.89)	19.57 (11.19, 27.95)
	female	27.60 (27.54, 27.66)	20.27 (20.22, 20.31)	17.76 (10.90, 24.62)

ASPR: age-standardized prevalence rate; CI: confidence interval; DS: Down syndrome.

## Discussion

As a globally prevalent chromosomal disorder, DS represents the leading genetic cause of intellectual disability (17). Individuals with DS face elevated risks for a range of comorbidities; congenital heart defects occur in approximately 40–50% of cases, and leukemia, immune disorders, and early-onset Alzheimer’s disease are also more prevalent (18–20). The clinical presentation varies widely, which can affect quality of life and place burdens on families and society (21). This study outlines the trends in the disease burden of DS in China and globally from 1990 to 2021. The results reveal a decline in the disease burden of DS in both China and the world. China has made greater progress than the global levels in reducing ASMR and ASDR related to developmental disorders (11). However, the ASPR and ASIR in China remain higher than the global levels. The changes in disease burden indicators over different periods reflect the progress of DS prevention and control strategies in China and globally. The correlation analysis between disease burden indicators and SDI highlights the key role of socioeconomic development in reducing the burden of DS. Forecasting analysis indicates that the disease burden of DS in China and globally will continue to decline in the long term.

Several key risk factors are associated with DS, with advanced maternal age being the most well-established non-modifiable factor. Socioeconomic trends such as delayed childbearing, particularly following China’s universal two-child policy in 2016, have increased population-level exposure to this risk (22). While the effects of paternal age remain less clear (23), modifiable factors include environmental exposures such as radiation, pesticides, air pollution (24), and endocrine-disrupting chemicals, which may interact with genetic predispositions and influence DS risk. However, the exact mechanisms and magnitude of these environmental risks remain uncertain due to limited epidemiological evidence and potential confounders. Lifestyle factors such as smoking, alcohol consumption, and nutritional deficiencies, especially folate deficiency (25), along with maternal metabolic disorders like diabetes and obesity and genetic predispositions, may compound DS susceptibility (26). Sociocultural disparities further limit access to preventive care, as marginalized groups face barriers such as healthcare resource shortages and low public awareness, which hinder early detection (27, 28). Given the complexity and uncertain contributions of factors, DS screening programs and maternal healthcare interventions are critical to reducing the disease burden. Prenatal DS screening has advanced remarkably in recent decades, evolving from traditional serological screening methods to include sophisticated molecular genetic techniques such as non-invasive prenatal testing based on next-generation sequencing (29). Second-trimester serological screening like the double or triple tests, identifies biochemical markers in maternal serum— namely alpha-fetoprotein, free beta-human chorionic gonadotropin, and unconjugated estriol— and combines these with maternal age and gestational age to estimate DS risk (30). The ultrasound measurement of nuchal translucency, which assesses fetal neck transparency thickness during 11–13^+6^ weeks of gestation, gained widespread adoption post-2000 (31). When integrated with serological indicators like pregnancy-associated plasma protein-A, this early combined screening achieves a DS detection rate of 80–85% (32). Non-invasive prenatal testing, leveraging high-throughput sequencing of cell-free fetal DNA in maternal blood to detect trisomies 21, 18, and 13 (33), demonstrates a DS detection rate exceeding 99% with a false positive rate below 0.1% (34).

Advances in the effective implementation of serological and molecular genetic testing have globally reduced the prevalence and incidence of DS (35). China exhibited a distinct pattern in DS epidemiology from 1995 to 2015, marked by a decisive shift after 2010. Initially, between 1995 and 2006, enhanced detection capabilities (ultrasound, genetic testing) paradoxically increased China’s ASPR, contrasting with the global decline. However, this trend reversed significantly between 2010 and 2015. During this period (2012–2015), China achieved a more rapid decline in its ASIR compared to the global average. This reversal and accelerated ASIR decline were driven by synergistic policy actions: the implementation of the national free preconception health examination project in 2010 and the catalytic release of the China birth defects prevention report in 2012 (36). These interventions fundamentally reshaped the trajectory: scaled prenatal genetic screening programs, enabled by the nationwide project, accelerated the ASIR decline. Simultaneously, systematic integration of these screenings with improved access to termination following diagnosis transformed enhanced diagnostic capabilities into sustained ASPR reduction by 2015. This combination brought China’s DS trends into closer alignment with those observed in high-income nations (37). Sociodemographic factors have shaped DS trends in China through multiple pathways (38). Although the ASIR continued to decline from 2015 to 2021, its deceleration coincided with an increase in advanced maternal age (≥35 years) following the introduction of the universal two-child policy in 2016 (39). This demographic shift elevated baseline DS risks, partially offsetting the preventive benefits of prenatal screening.

Between 1990 and 2021, cross-regional comparisons across the 21 GBD regions showed a positive correlation between ASIR/ASPR and SDI (40). This means that higher-SDI regions generally had higher overall DS incidence and prevalence. This phenomenon can be attributed to advancements in medical technology, changes in reproductive behaviors, and variations in data quality. High-SDI countries benefit from advanced prenatal screening technologies and standardized medical reporting systems, enabling them to accurately identify and report DS cases, which reflects higher statistics. Conversely, low-SDI regions face limitations in screening capabilities and weak data collection systems, causing underdiagnosis and underreporting, which consequently underestimate the true disease burden. In high-SDI countries, there is an increasing trend of women delaying childbearing until they are ≥35 years old (41), theoretically elevating DS incidence rates due to the exponential increase in DS risk associated with maternal age (42, 43). These seemingly contradictory patterns across and within regions highlight that the apparent advantages in data accuracy for high-SDI nations stem from both real risk factors and superior surveillance capabilities. Meanwhile, statistical biases in low-SDI regions mask unrecognized disease burdens.

Our analysis revealed inverse relationships between DS-related disease burden metrics (ASDR and ASMR) and SDI. High-SDI regions demonstrated substantially lower ASDR in 2021 compared to low-SDI regions, as well as a markedly reduced ASMR. Conversely, SDI showed positive correlations with ASPR and ASIR. These findings reflect two key mechanisms. Firstly, the advanced genetic testing capabilities have reduced birth defects in high SDI areas (44). Secondly, the enhanced medical interventions have lowered mortality rates while extending survival through prenatal screening, neonatal care, and rehabilitation support (45). China’s progress surpassed global trends between 1990 and 2021, achieving a 60% reduction in ASDR compared to the global decrease of 23%. Additionally, China’s ASMR declined by 63%, contrasting with the global reduction of 18%. By 2021, China’s ASDR approached high-middle SDI region averages, reflecting progress through public health investments and policies such as the National Free Preconception Health Check Program (2010) and the “Healthy China 2030” blueprint (2016). The latter explicitly called for strengthening the national birth defect prevention system and promoting screening and interventions across preconception, prenatal, and neonatal stages (46). These initiatives contributed significantly to earlier detection and improved management of DS, supporting the sustained decline in both ASDR and ASMR.

Epidemiological data indicate potential sex-based variations in DS distribution. A study of live-born infants with DS in Italy’s Tuscany region reported a male-to-female ratio of 1.3:1 (47). The study also noted that among DS patients with congenital heart defects, females exhibited a higher prevalence of atrioventricular septal defect, with approximately twice the risk observed in males. Analyses using the BAPC model of observed data and future projections demonstrated a consistent male predominance in the ASPR of DS globally and in China, highlighting potential sex-based disparities in disease burden. These findings collectively suggest that sex influences both clinical manifestations and population-level disease distribution in DS.

To sum up, based on high-quality GBD data, this study analyzed DS disease burden trends in China and globally from 1990 to 2021, exploring the links between policy interventions, SDI, and DS-related burden indicators. While the GBD provides robust global estimates, its limitations necessitate critical scrutiny, particularly with regard to underreporting in low-SDI regions. In these settings, inadequate healthcare infrastructure, shortages of trained personnel, and limited diagnostic access likely cause substantial underreporting and misclassification of DS cases. Consequently, the true disease burden may be underestimated, skewing regional and global interpretations. Future studies must integrate alternative data sources and include validation with independent real-world patient cohorts to address these limitations. Additionally, the lack of risk attribution meant this study could not analyze risk factors, while the absence of age-stratified incidence/mortality data for DS in GBD 2021 prevented the projection of trends using the BAPC method.

## Conclusion

Between 1990 and 2021, there was a reduction in the disease burden of DS in both China and around the world. The decline in China’s ASMR and ASDR outpaced the global average. Nevertheless, China’s ASIR and ASPR remained elevated. To address these persisting challenges and enhance healthcare outcomes, we recommend strengthening comprehensive prenatal assessments and healthcare services. These efforts should uphold strong ethical principles, promote informed decision-making, and ensure equitable, compassionate care.

## Data Availability

The datasets presented in this study can be found in online repositories. The names of the repository/repositories and accession number(s) can be found in the article/Supplementary material.

## Footnotes

^1^ https://ghdx.healthdata.org/gbd-2021

^2^ https://www.healthdata.org/research-analysis/about-gbd/protocol
